# Supplementary material for: Upregulated Ca2+ Release from the Endoplasmic Reticulum Leads to Impaired Presynaptic Function in Familial Alzheimer’s Disease
Source: Cells. 2022 Jul 11;11(14):2167. doi: 10.3390/cells11142167 (PMC9315668; doi:10.3390/cells11142167)
Supplement: Supplementary file 1 [file cells-11-02167-s001.zip › cells-1736094-supplementary.pdf]

Supplementary Figures

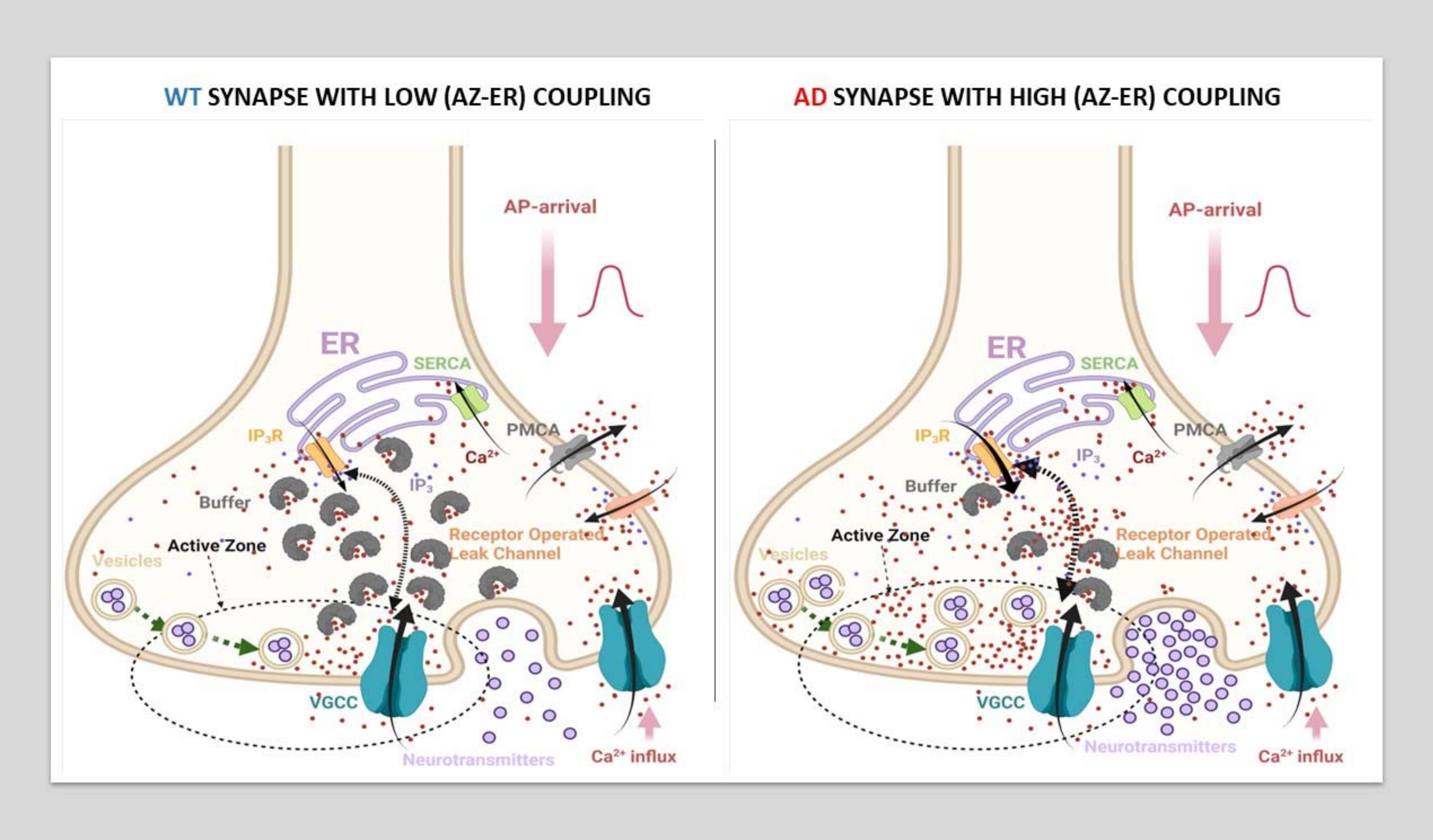

Figure S1. *Ca^{2+} homeostasis and neurotransmission scheme in FAD and WT synapses*. WT synapse (Left) with low ER-AZ coupling (thin dashed arrows). high buffering (grey half annuli), moderate AZ Ca^{2+} concentration, neurotransmission, and IP_3R activity (thin arrows), and FAD synapse (Right) with high ER-AZ coupling (thick dashed arrows), low buffering, elevated Ca^{2+} concentration, dysregulated and initially elevated release of neurotransmitters (purple) into the synapse, and IP_3R activity (thick arrows). *Ca^{2+}* is represented by small red circles and IP_3 by small blue ones.

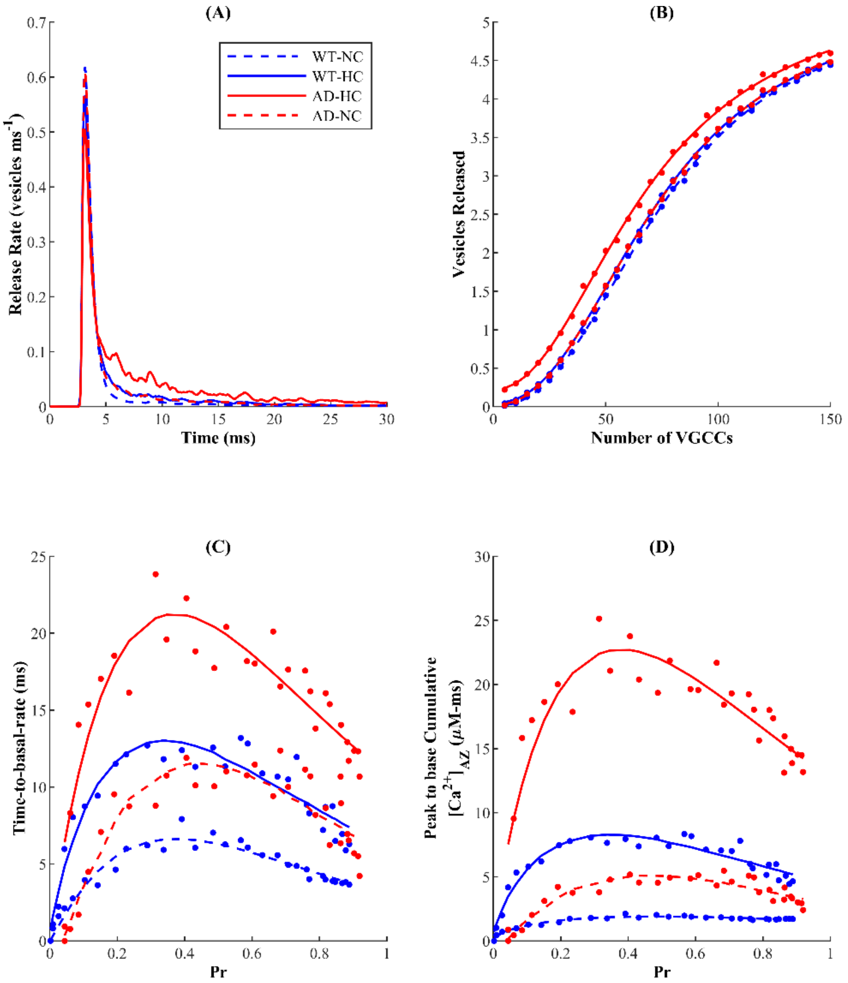

Figure S2. *Coupling between the microdomains around IP_3Rs and VGCCs influences Ca^{2+} in the AZ and neurotransmission profiles in FAD and WT synapses*. Transmitter release rates within 30 ms of stimulus (A) and total vesicles released (B) for different coupling configurations. Decay time of peak release rate (C) and cumulative Ca^{2+} concentration from peak to basal rate in the AZ (D) are markedly influenced by coupling.

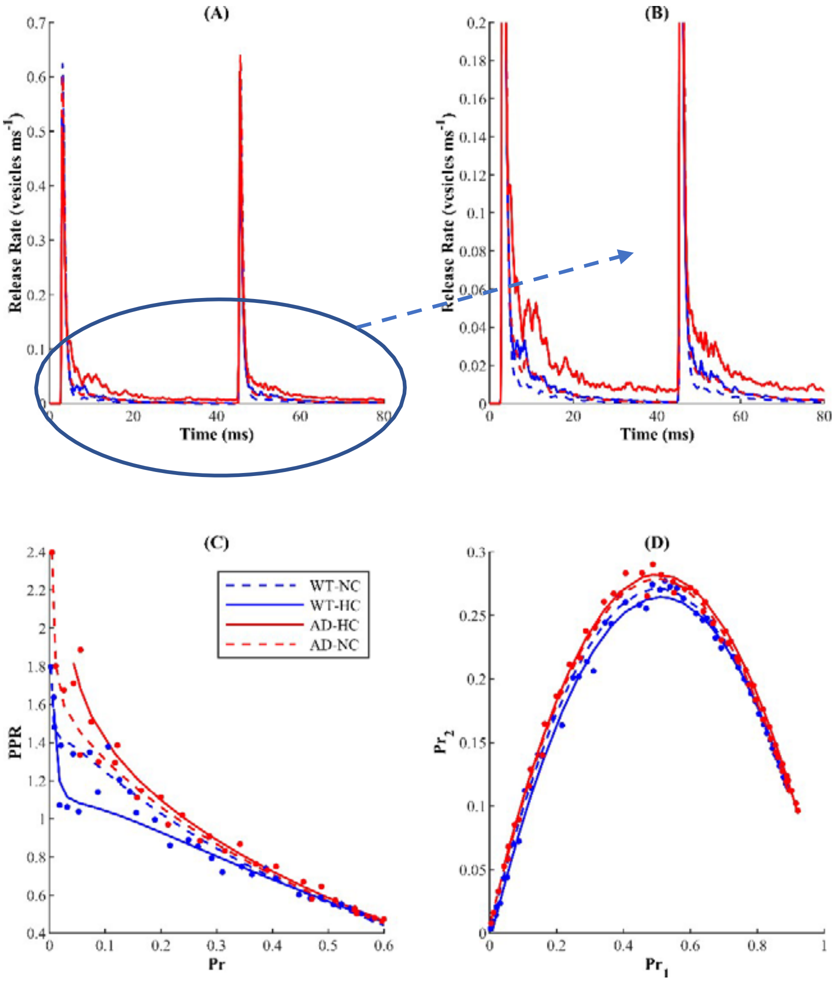

Figure S3. *Stronger coupling between the microdomian of IP3Rs cluster and AZ exacerbate the release rate and enhanced PPR in FAD-affected synapses but only marginally affect the bell-shaped behavior of Pr_2 as a function of Pr_1***.** Release profile (A) (zoomed-in (B)) following paired-pulse stimulation protocol shows an increase in release rate by stronger coupling in the microdomian of IP_3Rs cluster and AZ in both WT and FAD-affected synapses. (C) The enhanced PPR in FAD-affected synapses with respect to WT synapses is exacerbated by stronger coupling. (D) Pr in response to the second pulse ($Pr_{\#}$) as a function of Pr following the first pulse ($Pr_{\$}$) shows that the bell-shaped response is marginally affected by the coupling strength.

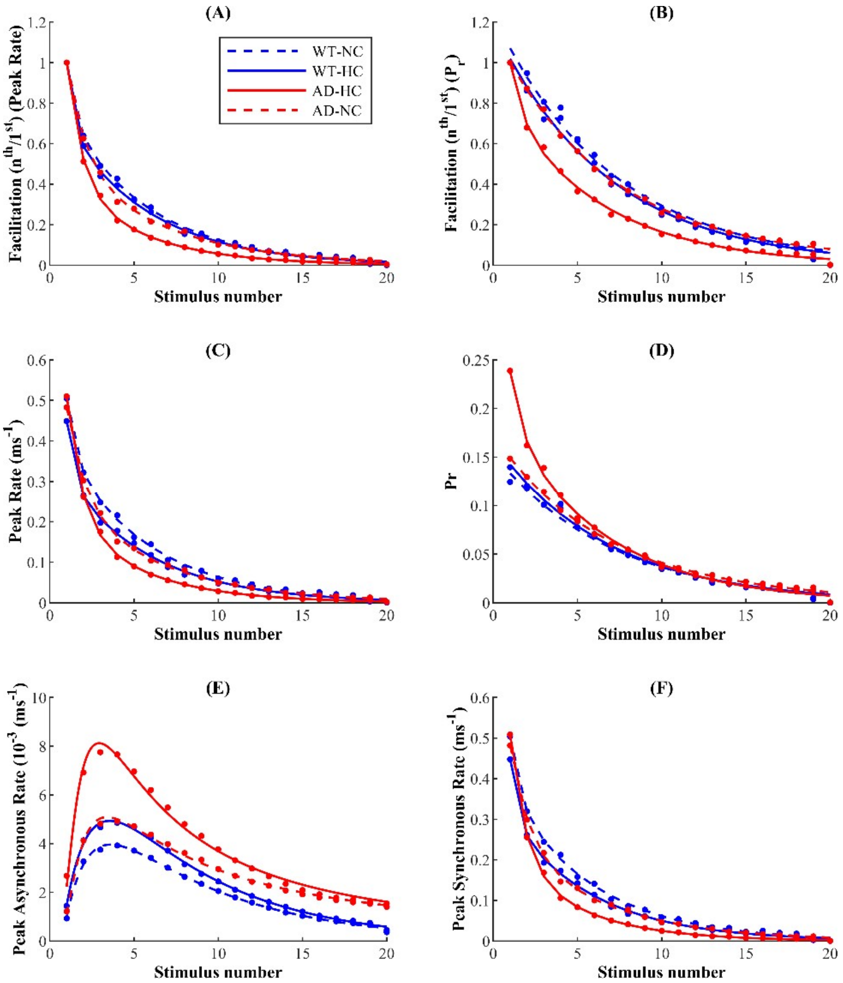

Figure S4. *Stronger coupling between the microdomian of IP3Rs cluster and AZ exacerbate the stronger depression in FAD-affected synapses*. Facilitation obtained from peak rate (A) and Pr (B) shows that HC enhances the synaptic depression in FAD-affected synapses. Peak release rate (C) and Pr (D) following each AP in the train under different coupling conditions. (E) Asynchronous and (F) peak synchronous release under different coupling conditions.
